# Considerations for increasing the competences and capacities of the public health workforce: assessing the training needs of public health workers in Texas

**DOI:** 10.1186/1478-4491-4-18

**Published:** 2006-07-26

**Authors:** Stephen Borders, Craig Blakely, Barbara Quiram, Kenneth McLeroy

**Affiliations:** 1 Grand Valley State University, Grand Rapids, Michigan, USA; 2 Prior Affiliation: Texas A&M University System Health Science Center – School of Rural Public Health, College Station, Texas, USA; 3 Texas A&M University System Health Science Center – School of Rural Public Health, College Station, Texas, USA

## Abstract

**Background:**

Over the last two decades, concern has been expressed about the readiness of the public health workforce to adequately address the scientific, technological, social, political and economic challenges facing the field. A 1988 report from the Institute of Medicine (IOM) served as a catalyst for the re-examination of the public health workforce. The IOM's call to increase the relevance of public health education and training prompted a renewed effort to identify competences needed by public health personnel and the organizations that employ them.

**Methods:**

A recent evaluation sought to address the role of the 10 essential public health services in job services among the Texas public health workforce. Additionally, the evaluation examined the Texas public health workforce's need for training in the 10 essential public health services.

**Results and conclusion:**

Overall, the level of perceived training needs varied dramatically by job category and health department type. When comparing aggregate training needs, public health workers with greater day-to-day contact (nurses, health educators) indicated a greater need for training than their peers who did not, such as those working in administrative positions. When prioritizing and designing future training modules regarding the 10 essential public health services, trainers should consider the effects of job function, location and contact with the public.

## Background

Over the last two decades, a series of national reports have expressed concern with the readiness of the public health workforce to adequately address the scientific, technological, social, political and economic challenges facing the field. The 1988 report from the Institute of Medicine's (IOM) Committee for the Study of the Future of Public Health served as a catalyst for the re-examination of the public health infrastructure and the workforce in particular. The Committee's call to increase the relevance of public health education and training for public health practice prompted a renewed effort to identify the scientific, technical, managerial and leadership competences needed by public health personnel in the field and the organizations that employ them [1]. In 2003, the IOM reiterated its call for action to train public health workers in core competences, specifically those working in public health departments [2].

Despite impressive gains over the past decades, many public health challenges remain. Changes in communicable disease control (HIV/AIDS, tuberculosis) and the evolution of social and behavioural problems (violence, addiction, obesity) have precipitated a renewed focus on population-based approaches to solving these problems [3-5]. Beaglehole and Dal Poz found that, in spite of the best efforts of academicians and policy-makers since the 1998 IOM report, little has been done and the "current organization and delivery of public health services are inadequate for these new challenges" [6]. These sentiments are shared by other experts as well, with Rowitz noting that the curriculum taught in many public health programmes may no longer be sufficient or effective in meeting future demands [7]. To meet the challenges of current public health problems, national leaders in the United States have encouraged local health departments (LHDs) to reduce direct-care, personal health services and focus more intentionally on population-based approaches to protect and promote health and to prevent disease and injury [1,4].

Since the 1988 IOM report, a number of researchers have sought to assess the education needs of the public health workforce. Several recent studies have addressed these concerns. One report addressed current deficits in the training of health educators. Allegrante et al. posed the question: "What are the skills that currently employed personnel need that they do not have?" and identified eight areas of competence and skill that were lacking [8].

A second study, conducted by researchers from the Pennsylvania and Northeast Public Health Workforce Training Project, identified universal competences and training priorities for the public health workforce in Maine, New Jersey, Pennsylvania, Rhode Island and Vermont [9]. A third study – of public health workers in Alabama, Arkansas, Louisiana and Mississippi-suggested a strong need for training in essential public health services [10].

More recently, public health workforce preparedness has taken on new urgency in response to the 2001 terrorist attacks, anthrax being sent through the United States postal system, the SARS outbreak and the spread of bird flu. An assessment of the public health workforce in North Carolina included an evaluation of core public health competences and emergency preparedness [11]. A study in Georgia focused exclusively on understanding the learning needs of the public health workforce as related to bioterrorism and emerging health threats, establishing baseline data for evaluating future training programmes [12].

At the national level, an assessment of the training needs of LHD executive administrators provided a direct measure of the backgrounds of key administrators at formal local public health agencies across the country. In general, they found that nearly 80% of the respondents had no formal public health training [13].

Prior to this study, a group of Texas researchers examined characteristics of the public health workforce in Texas [14]. Kennedy et al. developed a two-stage sample survey to estimate the size of the workforce in the state and to describe settings as well as job and personnel characteristics. They concluded by raising concerns about the adequacy of the formal education of the public health workforce in Texas, providing the catalyst for the research described here.

Despite the flurry of research in the area over the past years, a number of important questions have been insufficiently explored: First, which types of public health workers need additional training in public competences? For example, do nurses and health educators have the same training needs? Second, do training needs vary by public health setting? For example, do public health workers employed by the state health department have the same training needs as those employed in LHDs?

### Defining the public health workforce

Effective training and education for a continuously evolving workforce and field, such as public health, require a clear understanding of the composition, nature and services of the workforce. However, there is little consistency among public health worker job definitions at the national or local level. The public health workforce has been defined as "those individuals employed by local, state, and federal government health agencies" [15]. Still others contend that the public health workforce should include a wider array of workers to include those in academia, private sector employees who provide community-based services and education and economic development professionals [15].

The difficulties of defining the public health workforce are further compounded by a lack of standardization among job categories. The Bureau of Labor Statistics at the United States Department of Labor tracks the nation's workforce and classifies job categories through a scheme called the Standard Occupational Classifications system. The system classifies workers in a four-tiered pyramid fashion, starting with broad major groups and ending with a detailed description of the occupation [16]. Gerzoff and Gebbie note that despite the millions currently employed in public health, none of the detailed categories in the Standard Occupational Classifications system are specific to public health; they recommend more rigorous definitions [7]. Although the Standard Occupational Classifications system provides some direction in classifying job categories, the diversity and ambiguity among the public health workforce both nationally and in Texas defies simple aggregation.

### Employment settings

Employment settings are critically important in assessing the public health workforce because of the interaction with the public. Health departments in Texas vary widely in their size, functionality and services offered. This variability often dictates the level and kind of interaction with the public. Employees of LHDs are typically the visible presence of the public health workforce. LHD workers typically provide public health services such as immunizations, STD treatment and restaurant inspections, and are viewed as the first-line responders to health emergencies.

Texas has two types of LHDs. Currently, there are 67 LHDs in Texas that receive state funding [17]. Because of this state funding, these health departments are referred to as "participating" health departments. The state funds comprise two sources: approximately 25% from the Preventive Health and Human Services (PHHS) block grant from the Centers for Disease Control and the remaining 75% from state general revenue [18]. By receiving state funds, participating LHDs have certain requirements and services they must provide as a condition of receiving that funding. As a result, participating LHDs most often provide a wide array of public health services, such as immunizations, restaurant and septic tank inspections, maternal and child health care services, public health education, dental services and HIV and STD counseling.

There are 78 LHDs that receive no state funding and are called "non-participating" LHDs [19]. Several of these non-participating LHDs are large, full-service health departments, but most are small and provide mainly environmental services such as animal control and septic tank and restaurant inspections. Non-participating health departments do not receive state funds or assistance, but are still eligible for certain federal funds.

In addition to LHDs, the State of Texas operates two types of state-level health departments because of the rural nature and sparse population in many of Texas' counties. While the state-level health departments do provide some direct services to the public, they are typically more engaged in broad policy-setting and administrative functions. In addition, the state-level health departments also provide support services for LHDs.

The State of Texas is divided into 11 public health regions. The public health regions were established in 1970 as the legislature recognized the necessity for complete public health services to be made available to all the people in all Texas counties. Prior to that time, only 67 of Texas' 254 counties had organized public health services. The intent of the legislative agenda behind the creation of regional public health offices was to concentrate a collection of public health professionals and special consultants in a central location where their expertise could be used efficiently by counties with and without organized health units.

The regional departments support all health programmes, provide comprehensive public health services and provide assistance to the other organized health units within the region. The public health regions are extensions of the Texas Department of Health, now known as the Texas Department of State Health Services (TDSHS) and operate under the Commissioner of Health [20]. Today there are eight regional public health headquarters around the state, as some regions share administration of more than one public health region These regional health departments often act as the sole public health presence in many of Texas' most rural counties [21]. In addition to the regional health departments, the state's primary health department has its headquarters in Austin, serving as the lead agency for administering and setting policy for the state's public health programmes.

### The Texas Public Health Training Center

In 2000, the School of Rural Public Health at the Texas A&M University System Health Science Center, the School of Public Health at the University of North Texas Health Science Center and the School of Public Health at the University of Texas-Houston established the Texas Public Health Training Center (TPHTC). The mission of the TPHTC is to ensure that the Texas public health workforce has access to high quality learning programmes as a means of strengthening technical, scientific, managerial and leadership competences and capabilities of current and future public health workers.

Funding for the TPHTC was established under P.L. 105–392, the Health Professions Education Partnerships Act of 1998, to assess the learning needs of the public health workforce and provide training to meet them. Currently, 44 states and the District of Columbia are covered by similar Public Health Training Centers. Core funding comes from the United States Department of Health and Human Services through the Health Resources and Services Administration – Bureau of Health Professions, which seeks to improve the nation's public health system by strengthening the technical, scientific, managerial and leadership skills and abilities of current and future public health professionals [22].

The development of the TPHTC was projected in a four-phase plan, scheduled to be completed over a five-year project period. The results reported in this article reflect Phase I objectives. One of the primary objectives of Phase I was to conduct a needs assessment of the Texas public health workforce. This objective was directly influenced by House Bill 1444, passed in the 76th Texas Legislature in 1999.

The Bill was the culmination of two years of study conducted by the TDSHS, the School of Public Health at the University of Texas-Houston, the LBJ School of Public Affairs at the University of Texas and the School of Rural Public Health at the Texas A&M University System Health Science Center. As a result of that work, Texas became the first state in the United States to have the 10 essential public health services specifically written into the state statute, Local Public Health Services Act – Health and Safety Code 121.0021. The 10 essential public health services are as follows:

• monitor health status to identify community health problems;

• diagnose and investigate health problems and health hazards in the community;

• inform, educate and empower people about health issues;

• mobilize community partnerships to identify and solve health problems;

• develop policies and plans that support individual and community health efforts;

• enforce laws and regulations that protect health and ensure safety;

• link people to needed personal health services;

• assure a competent public health and personal health care workforce;

• evaluate effectiveness, accessibility, and quality of personal and population-based health services;

• research new insights and innovative solutions to health problems.

## Methodology

The instruments, sample design and survey methodology were designed in collaboration with members from the TPHTC and leaders within the TDSHS. The result was the development of an instrument to assess two primary objectives: the role the 10 essential public health services play in the job services among the Texas public health workforce; and the Texas public health workforce's need for training in the 10 essential public health services.

### The public health workforce subjects and sample

To build a sampling frame from which to target members of the public health workforce who are actually employed in the Texas public health system, we began by examining system-defined categories of existing personnel at participating LHDs, non-participating LHDs and at the TDSHS central office in Austin and TDSHS regional offices. Initial tallies of data gleaned from the directors of these public health agencies indicated that approximately 5250 individuals are employed at the central and regional TDSHS offices. Another 13 350 are employed in Texas at participating LHDs and 600 are employed at non-participating LHDs.

Through analysing the particular job services for the various types of public health providers from job descriptions provided by TDSHS, a list of job categories was developed to sample (see Table 1). This process also helped to eliminate health department employees whose job services appeared outside the realm of public health practice, such as receptionists, administrative assistants, physical plant support and other employees who typically do not deliver public health services. The categories of "manager or administrator at TDSHS" and "public health technician or other" were added after the original job categories were created. These categories were created because of the difficulty in placing these types of employees into other functional job categories and the non-descriptive nature of their job titles.

**Table 1:** Job categories of the Texas public health workforce

**Job category**	**Examples of job titles**
WIC/Nutritionists	Those associated with the Women, Infants and Children's Programme: Nutritionist, WIC Counselor, WIC Supervisor
Nurses	Licensed Nurses: LVN, BSN
Sanitarians	Local inspectors: Restaurant Inspector
Dental Workers	Dental assistants, dental hygienists, but not dentists
Case and Social Workers	Caseworker, clinical social worker, community health service aid
Lab	Microbiologists, lab workers
Licensed Health Professionals	Pharmacists, Physicians, Veterinarian, Dentists
Animal/Vector Control	Animal control, mosquito control
HIV/STD	HIV/STD prevention, risk management
Health Educator	Health Educator
Biostatisticians Epidemiologists	Biostatistician, epidemiologist
Public Health Technician and other	Public Health Technician, Programme Specialist (job categories within the TDSHS that were non-descriptive) and other
Manager or Administrator at TDSHS	Manager, Director at TDSHS central office in Austin or a TDSHS regional health department
Manager or Administrator at LHD	Administrative staff other than the director at a local health department: Manager, Health Services Coordinator, Director of Programmes

In the summer of 2002, the Texas A&M University System Health Science Center's School of Rural Public Health solicited the assistance of each health department from around the State. Directors of LHDs were asked to provide the names and contact information for their employees that worked in each of the job categories. Unfortunately, not all LHD department directors in the state of Texas responded to the request for information. Participating LHD compliance was much better than non-participating local health department compliance. Approximately half of the 67 participating LHDs responded to the request for information, while slightly less than 25% of the 78 non-participating LHDs responded.

The data were received and entered into an interactive database, containing each public health employee's name, job title and mailing address at their place of work. Where possible, we obtained other contact information such as the employee's phone number and email address. The TDSHS central office in Austin and TDSHS regional health departments provided contact information through a database, which was then pared down to the relevant public health categories of interest for this study, excluding workers with job titles deemed outside the realm of public health practice.

### Procedure and measures

Surveys were mailed to each public health worker in the database (N = 5860), beginning in September of 2002. Each member of the public health workforce received a survey and cover letter explaining the purpose of the research and the questionnaire. A return, postage-paid envelope was provided for convenience; all completed questionnaires were returned to the Public Policy Research Institute at Texas A&M University (PPRI) for data entry. PPRI is an applied, policy-relevant research organization with an extensive survey laboratory that provides scientific research, data collection and evaluation services to both public and private sponsors. To boost participation rates, a follow-up postcard reminder was mailed out approximately 10 business days after the mailing of the questionnaire; it provided a toll-free number and email address for questions or concerns.

Results from the initial wave of direct surveys were disappointing. The methods were costly in several terms, including creating the database, data entry, disseminating follow-up reminders and mailing costs. With limited survey funds remaining, we randomly sampled 900 public health workers who had not completed a questionnaire as of November of 2002 for a second mailing. The second wave of questionnaires followed the same protocol as the first wave and led to a measurable improvement in the number of completed surveys.

Additional reminders via email and telephone were sent to TDSHS management and LHD directors to encourage participation among their employees. The final completion rate (see Table 2) for the study was 31% (1812 surveys), which is within norms of similar types of mail surveys [23-25].

**Table 2:** Completion rates (number in the sample/percent of sample responding) by public health worker category and health department type

**Public health worker category**	**Participating**	**Non-participating**	**TDSHS regions**	**TDSHS central office**	**Overall completion rate**
Nutritionists/WIC	276/.34	0/0	26/.69	17/.29	319/.37
Nurses	570/.30	7/.71	197/.51	138/.18	911/.33
Sanitarians	285/.28	112/.34	355/.35	142/.29	895/.32
Dental Workers	56/.32	7/0	31/.29	5/.40	95/.31
Social/Case Worker	358/.27	7/.43	249/.43	19/.21	633/.33
Lab	100/.35	0/0	0/0	237/.26	337/.29
Licensed Health Care Professionals	84/.19	5/.20	13/.46	59/.22	161/.22
Animal/Vector Control	260/.23	53/.09	7/.71	0/0	320/.22
HIV/STD	75/.40	0/0	3/0	0/0	78/.38
Health Educators	92/.36	2/.50	4/.40	0/0	99/.36
Biostatisticians Epidemiologists	37/.62	0/0	10/.70	46/.65	93/.65
Public Health Tech/Other	257/.14	0/0	496/.39	649/.24	1420/.28
Manager or Administrator at TDSHS	0/0	0/0	63/.41	344/.22	406/.25
Manager or Administrator at LHD	105/.50	6/.83	0/0	0/0	111/.51
**Total**	**2555/.29**	**200/.29**	**4455/.41**	**1656/.25**	**5860/.31**

The survey was specifically designed to elicit detailed responses from the Texas public health workforce regarding their personal level of need for training on all core public health competences. Survey items were constructed on the basis of face validity; building on a previous instrument adopted from a similar needs assessment used by Tulane University for the public health workforce in Alabama, Arkansas, Louisiana and Mississippi [10].

Respondents were asked five questions related to their need for additional training in each of the 10 essential public health services. For each item, the public health workforce member responded on a five-point Likert scale with an agreement continuum. Individuals self-assessed the importance of each activity to his or her job on a scale of 1 (not necessary) to 5 (critically necessary). Respondents were also asked to provide basic demographic information, as well as their educational levels and experience in public health programmes. Table 3 provides an example of the questionnaire structure for items associated with the first essential public health service: "monitor health status to identify community health problems".

**Table 3:** Example of an essential public health service domain for the Public Health Workforce Questionnaire

**Do you need training?**	**not necessary – critically necessary**				
Use epidemiology to monitor health status, identify community and public health problems.	1	2	3	4	5
Use epidemiology to identify community health problems through surveillance strategies.	1	2	3	4	5
Oversee a community assessment process to identify community health problems	1	2	3	4	5
Apply risk assessment techniques to identify community health problems	1	2	3	4	5
Assessment of health service systems in order to identify community health problems	1	2	3	4	5

The data reported in this article represent respondents' perceived need of training for each of the 10 essential public health services. A complete description of the full results, including the survey instrument, cover letter, directions and sampling is available in a technical report [26].

## Results

Public health workers from participating LHDs made up the largest portion of respondents with 41%, followed by TDSHS regional staff with 32% responding, TDSHS central office staff with 24 % and non-participating local health department staff with 3% responding. The majority of respondents were females (70%), who were also white (60%) and between the ages of 46 and 55 (39%). A list of selected demographic variables can be found in Table 4.

**Table 4:** Descriptive statistical summaries (number in the sample/percent of sample responding) for selected demographic variables by health department type and for the total sample (n = 1745)

Item	**Participating LHD (n = 741)**	**Non-participating LHD (n = 58)**	**TDSHS Regions (n = 599)**	**TDSHS Central Office (n = 414)**	**Total**
**Gender**					

Male	541/.77	21/.38	397/.68	261/.65	1220/.70
Female	165/.23	34/.62	183/.32	143/.35	525/.30

**Current age**					

24 or younger	36/.05	2/.21	5/.01	11/.03	54/.03
25–34	141/.20	12/.21	83/.14	62/.15	298/.17
35–44	172/.24	19/.33	167/.29	129/.32	487/.28
45–54	255/.36	19/.33	245/.42	161/.40	680/.39
55 and older	100/.14	5/.09	78/.13	40/.10	223/.13

**Ethnicity**					

African American or Black	151/.22	5/.09	39/.07	32/.08	227/.13
Asian	22/.03	1/.02	6/.01	9/.02	38/.02
Hispanic or Latino	128/.18	9/.16	178/.31	54/.14	369/.21
Native American	4/.01	0/0	3/.01	3/.01	10/.01
White	379/.54	41/.72	333/.59	288/.73	1041/.61
Other	13/.02	1/.02	9/.02	11/.03	34/.02

**Total years in current position**					

0 to 5	393/.56	38/.69	283/.49	254/.62	968/.55
6 to 10	161/.23	7/.13	158/.27	88/.22	414/.24
11 to 15	88/.12	4/.07	72/.12	34/.08	198/.11
16 to 20	28/.04	4/.07	25/.04	20/.05	77/.04
21 or more	37/.05	2/.04	44/.08	13/.03	96/.05

**Total years in public health**					

0 to 5	268/.38	25/.44	121/.21	103/.25	517/.29
6 to 10	165/.23	10/.18	157/.27	92/.23	424/.24
11 to 15	117/.16	3/.05	127/.22	75/.18	322/.18
16 to 20	62/.09	11/.19	75/.13	65/.16	213/.12
21 or more	99/.14	8/.14	109/.19	71/.17	287/16

**Highest level of education**					

High school	105/.15	7/.12	101/.18	38/.09	251/.14
Associate/Technical degree	133/.19	13/.23	152/.27	43/.11	341/.20
Master's degree	288/.41	24/.42	213/.37	165/.41	690/.40
Master's degree	137/.20	12/.21	85/.15	113/.28	347/.20
Doctoral degree	37/.05	1/.02	20/.04	45/.11	103/.06

**Graduate degree in public health**					

No	656/.94	51/.93	550/.97	363/.90	1620/.94
Yes	43/.06	4/.07	18/.03	40/.10	105/.06

Overall, the public health workers who responded to the survey have attained high levels of education. The majority of respondents (66%) had at least a college degree. Twenty-six percent of the respondents had advanced degrees, such as master's or doctorate degrees. Despite having advanced degrees, only 6% indicated having a graduate degree in public health

After some preliminary analysis within each of the 10 essential public health categories, the data appeared to be highly correlated within each of group of questions. For example, respondents tended to rate each of the five questions within the general battery of questions related to the essential public health function – monitoring health status to identify community health problems similarly. Reliability analysis showed that items within each of the 10 essential public health functional areas had high internal consistency [27].

To aid in the data analysis, we assigned numbers to ranks, essentially converting ordinal to interval data [28-30]. Because the data were highly correlated and this method of data reduction provided a clearer means for interpreting the results, the responses to each of the 10 essential public service categories were pooled to create an overall mean for ranking and comparison on each of the functional areas. A mean closer to five indicates respondent need for training in the essential public health skill as critical. Conversely, a lower mean, closer to one, indicates that the respondents viewed additional training in the essential public health skill as unnecessary.

Using the responses from the questions in each public health category related to training needs, we developed an overall mean from the aggregated responses to determine "need" for additional training. Overall, public health worker job categories with a mean equal to or greater than 3 were determined to have a positive bias and thus a "need" for additional training in an essential public health area. Table 5 shows a summary of public health worker by job classification and their training needs within each public health area.

**Table 5:** Summary of public health workers indicating a need for training

**Job category**	**Mean**	**SD**	**Essential public health function**
WIC/Nutritionist (N = 104)	3.06	1.14	Inform, educate and empower people about health issues
Nurses (N = 271)	3.05	1.22	Monitor health status to identify community health problems
	3.15	1.20	Enforce laws and regulations that protect health and ensure safety
	3.13	1.21	Mobilize community partnerships to identify and solve health problems
	3.10	1.23	Develop policies and plans that support individual and community health efforts
Sanitarians (N = 256)	3.53	1.15	Enforce laws and regulations that protect health and ensure safety
	3.05	1.21	Inform, educate, and empower people about health issues
Animal and Vector Control (N = 59)	3.23	1.37	Enforce laws and regulations that protect health and ensure safety
	3.08	1.35	Inform, educate, and empower people about health issues
Health Educators (N = 34)	3.05	1.47	Evaluate effectiveness, accessibility, and quality of personal and population-based services
	3.03	1.09	Inform, educate, and empower people about health issues
	3.01	1.18	Develop policies and plans that support individual and community health efforts
	3.01	1.27	Mobilize community partnerships to identify and solve health problems
Biostatisticians and Epidemiologists (N = 57)	3.07	1.10	Inform, educate, and empower people about health issues
Administrators at TDSHS (N = 91)	3.08	1.20	Ensure a competent workforce
Administrators at local health departments (N = 49)	3.14	1.22	Develop policies and plans that support individual and community health efforts
	3.13	1.19	Inform, educate, and empower people about health issues
	3.05	1.20	Ensure a competent workforce

As a group, sanitarians indicated the single greatest need for training, with a focus on the essential public health service of enforcing laws and regulations that protect health and ensure safety. While sanitarians from all of the four health department types provided responses, those in participating (mean of 3.8) and non-participating (mean of 3.8) LHDs indicated a stronger need for training than did their counterparts in either the TDSHS central office (mean of 3.4) and TDSHS regional departments (mean of 3.3). Nurses and health educators expressed the greatest need for training across a variety of the essential public health services. Both groups of workers shared similar sentiments for training needs in three of the essential service areas: informing, educating and empowering people about health care issues; mobilizing community partnerships; and developing policies and plans that support health efforts.

As a group, public health workers consistently rated two essential public health themes among the top training needs: enforcing laws and regulations to protect health; and informing, educating, and empowering people about health issues. These groups of public health workers included nutritionists/WIC personnel, nurses, sanitarians, animal and vector control workers, and biostatisticians and epidemiologists.

Due to variability within the public health worker categories across worker locations, we derived a single mean from the 10 essential public health services to determine an overall need for additional training. One-factor ANOVA comparisons of differences in the overall means depicting need for additional training found significant differences in the 10 essential public health services among the four health department types (*F*(3,1733) = 12.45, p < .001). Post hoc Tukey Significant Difference tests, which account for multiple comparisons, revealed that the overall need for training among employees working for the TDSHS central office in Austin was significantly lower than for the other three health department types. The mean need for additional training was significantly lower than TDSHS regional health departments (p < .001), non-participating LHDs (p < .05) and participating LHDs (p < .001). These findings seem to indicate that training in the 10 essential public health services is less of a priority at the TDSHS central office than for participating and non-participating LHDs and TDSHS regional health department workers.

## Discussion

The public health system must respond to continual changes in national, state and local health care systems, budgets and policies. As the demand for public health services both transforms and increases, so do the training needs of the public health workforce. Public health workers have demanding jobs that require advanced professional skills and expertise. It is clear that with an ever-changing public health system and complex public health problems locally, nationally and internationally, ongoing training must be an essential component of the public health system. Thus, several policy issues have emerged in response to the training needs of the public health workforce.

First, because of the diversity of both location and job role, coupled with the location of the respondents in this study, it is extremely important to prioritize and define the training for specific segments of the workforce. Indeed our results show that training needs do vary by public health setting. For example: public health workers at the TDSHS central office in Austin indicated less desire for additional training in the essential public health services than did workers in the regional offices and local health departments. The differences in the perceived training needs of these workers in varying roles and locales may be explained by differences in day-to-day personal contact with the general public. Those who tended to have more contact with the public indicated a greater need for training. Central office personnel (TDSHS-Austin) typically perform more administrative, support and policy-setting functions, while those in regional offices are more likely to be actively engaged with the public.

This finding regarding day-to-day personal contact was consistent with consideration of role or type of public health worker as well. Nurses, sanitarians and health educators, who are actively engaged with the public in daily work, expressed a stronger need for training in essential services.

However, one notable exception to this finding was those working in the area of HIV/STD. This finding suggests a crucial difference between public health workers who are generalists and those who are specialists. Generalists (nurses, health educators) typically move from disparate tasks throughout their workday, such as prenatal education to immunizations, while specialists (HIV/STD workers, epidemiologists) can often devote their knowledge and skills to a single public health area. Thus, generalists most likely need a wide range of skills and training in public health competences to address a number of changing and perhaps disparate conditions or issues, while specialists may not.

Specialists who indicated a lower need for training may also be influenced by the intensive training and education that many specialists receive as a part of or prior to working within the field. This may also be compounded by their ability to concentrate in a specific public health concern as a practitioner, thus avoiding the need to learn skills outside their original field of expertise or training.

These findings also support the work of the Council on Linkages Between Academia and Public Health Practice. The Council's mission is to improve public health practice and education by establishing links between academia and public health agencies. The group has developed guidelines for individuals who need to develop core public health competences. The skill levels differ for individuals, such as front-line workers versus those in management positions, ranging from those who need to be aware of specific public health competences to those needing proficiency [31]. As a result, when prioritizing and designing future training modules of the 10 essential public health services, any training considerations should consider the job categories of the public health workforce, the location in which the public health professionals work and the day-to-day contact each worker has with the public.

This study had several limitations. Although the research focused on what many consider to be the primary or "core" public health providers in Texas, it did not include the training needs of all public health workers in the state. The public health workforce in Texas is much broader, including employees of federal public health agencies and other governmental entities that house subsidiary units that provide public or environmental health services.

There are also a number of private, nonprofit associations or community-based organizations that focus on general or specific health problems in the context of larger social or economic issues. The public health workforce also includes organizations that provide personal health services, such as hospitals, outpatient facilities and long-term care facilities. Educational institutions, such as primary, secondary and post-secondary schools are also major settings for the provision of health and safety services [14].

Thus, more research is needed to determine the training needs of those members outside the "core" public health workforce. In addition, we reiterate Gerzoff and Gebbie's call for developing more rigorous definitions to permit a more reliable and effective enumeration of the public health workforce.

A second limitation is that the results from the low number of respondents from non-participating LHDs should be interpreted cautiously. The low response rates were most likely a function of two issues: non-participating public health employees comprised a lower number of the total potential subjects; fewer non-participating LHDs responded to our request for information when building our database, thus leaving a far smaller pool from which to sample.

This likely reflects a very real distinction about how different workers in these LHDs view their role in relation to providing essential public health services. Non-participating LHDs tend to be found in more rural settings, focusing almost exclusively on the delivery of historically "essential services" such as restaurant inspections and water quality, maintaining only limited involvement with the state health department. Thus, the role of non-participating LHDs in making policy and influencing practice has traditionally been limited.

Given these limitations, the study's results provide an approach for improving the public health workforce training and skills by identifying training topics in the essential public health services and the timing of such training. Because this study identified only the topics, curriculum development should also include appropriate pedagogic strategies for the diverse professional and educational backgrounds of the public health workforce.

For example, traditional classroom instruction may be more appropriate for public health workers in highly centralized systems such as the TDSHS central office in Austin. Internet-based or other forms of distance instruction may make more sense for public health workers in LHDs who may not have the time or the funds available to travel for training purposes. Other considerations might also include the value employers place on training, such as granting employees work release for educational and training activities.

In addition, this work further underscores the disparate backgrounds of many public health workers that begin far outside the field of public health. The majority of public health workers do not hold public health degrees such as a Master of Public Health [32], often regarded as the requisite degree for entry into the field. For new employees, core competence training should be a standard part of orientation training or should take place soon after the date of hiring to ensure that the public health workers have the requisite skills to effectively do their jobs.

## Competing interests

The author(s) declare that they have no competing interests.

## Authors' contributions

Stephen Borders was the principal author of the article. Each author substantially contributed to the research project and the article. Each was involved in the study design and data analysis. In addition, each of the authors reviewed and commented on the manuscript prior to its submission.

## References

[B1] Institute of Medicine (1998). The Future of Public Health.

[B2] Institute of Medicine (2003). Who will keep the public healthy? Educationg public health professional in the 21st century.

[B3] McMichael AJ, Beaglehole R (2000). The changing global context of public health. Lancet.

[B4] Baker EL, Melton RJ, Stange PV, Fields ML, Koplan JP, Guerra FA, Stacher D (1994). Health reform and the health of the public: Forging community health partnerships. JAMA.

[B5] Wall S (1998). Transformation in public health systems. Health Affairs.

[B6] Beaglehole R, Dal Poz M (2003). Public health workforce: challenges and policy issues. Human Resources for Health.

[B7] Gerzoff RB, Gebbie KM, Novick LF, Mays GP (2005). The Public Health Workforce. Public Health Administration: Principles for Populations-Based Management.

[B8] Allegrante JP, Moon RW, Auld ME, Gebbie KM (2001). Continuing-education needs of the currently employed public health education workforce. American Journal of Public Health.

[B9] Potter MA, Pistella CL, Fertman CI, Dato VM (2000). Needs assessment and a model agenda for training of the public health workforce. American Journal of Public Health.

[B10] Chauvin SW, Anderson AC, Bowdish BE (2001). Assessing the professional development needs of public health professionals. Journal of Public Health Management and Practice.

[B11] Harrison LM, Davis MV, MacDonald PD, Alexander LK, Cline JS, Alexander JG, Rothney EE, Rybka TP, Stevens RH (2005). Development and implementation of a public health workforce training needs assessment survey in North Carolina. Public Health Reports.

[B12] Childers W, Alperin M, Miner KR (2005). Emergency preparedness in Georgia: An Assessment of public health training needs. American Journal of Health Education.

[B13] Gerzoff RB, Richards TB (1997). The education of local health department top executives. Public Health Management Practice.

[B14] Kennedy VC, Spears WD, Loe HD, Moore FI (1999). Public health workforce information: A state-level study. Journal of Public Health Management and Practice.

[B15] U.S. Department of Health and Human Services (1997). The public health workforce: An agenda for the 21st century.

[B16] Department of Labor, Bureau of Labor Statistics (2000). Standard Occupational Classification.

[B17] Texas Department of Health Full Service Local Health Departments and Districts of Texas. http://www.dshs.state.tx.us/regions/lhds.shtm.

[B18] Texas Health and Human Services Comission (1998). The impact of Medicaid managed care on the public health sector.

[B19] Texas Department of Health Texas Non-participating Local Health Departments. http://www.dshs.state.tx.us/regions/nonlhd.shtm.

[B20] Texas Department of Health (1973). Biennial Report: September 1, 1970 - August 31, 1972.

[B21] Texas Department of Health Texas Public Health Regions. http://www.dshs.state.tx.us/regions/default.shtm.

[B22] Health Resources and Services Administration Public Health Training Centers. http://bhpr.hrsa.gov/publichealth/phtc.htm.

[B23] Cobanoglu C, Warde B, Moreo P (2001). A comparison of mail, fax, and web-based survey methods. International Journal of Market Research.

[B24] Harabaugh R (2002). Proven lessons for generating good mail response rates. Medical Marketing and Media.

[B25] Schuldt B, Totten J (1994). Electronic mail vs. mail survey response rates. Marketing Research.

[B26] Borders S, Blakely C (2002). The Ten Essential Public Health Services: Assessing the Training Needs of the Texas Public Health Workforce.

[B27] Cronbach LJ (1951). Coefficient alpha and the internal structure of tests. Psychometrika.

[B28] Labovitz S (1971). In defense of assigning numbers to ranks. American Sociological Review.

[B29] Labovitz S (1970). The assignment of numbers to rank order categories. American Sociological Review.

[B30] Nardi PM (2003). Doing survey research: A guide to quantitative methods..

[B31] The Council on Linkages Between Academia and Public Health Practice Core competencies for public health professionals: A practical tool to strengthen the public health workforce. http://www.trainingfinder.org/competencies/list.htm.

[B32] Brown GB, Humphrey B, Pallister R (1982). Prevelance and characteristics of frequent attenders in a prepaid Canadian family practice. Journal of Family Practice.

